# Utilization of blood by-products: An *in silico* and experimental combined study for BSA usage

**DOI:** 10.1038/s41598-017-17029-2

**Published:** 2017-12-08

**Authors:** Fátima Arrutia, Rebeca Fernández, Carlos Menéndez, Ulises A. González, Francisco A. Riera

**Affiliations:** 1 Chemical Engineering and Environmental Technology Department, University of Oviedo, Julián Clavería, 8, 33006 Oviedo, Asturias Spain; 2 Research Institute in Chemical Technology (INTEQUI)-CONICET, Faculty of Chemistry, Biochemistry and Pharmacy, Universidad Nacional de San Luis (UNSL), Ejército de los Andes 950, 5700 San Luis, Argentina

## Abstract

In order to exploit industrial discards, protein enzymatic hydrolysis is a currently popular methodology for obtaining bioactive peptides. However, once released, most promising peptides have to be selected from the mixture. In this work, the suitability of pepsin (EC 3.4.23.1) to hydrolyse serum albumin in order to obtain bioactive peptides was assessed. Then, a suitable process to obtain best separation of bioactive peptides was evaluated, using polyethersulfone membranes at different pH values. Serum albumin was easily hydrolysed by pepsin, reaching a DH value of the 65.64 ± 1.57% of the maximum possible. A 23.25% of the identified peptides possessed high bioactivity scores (greater than 0.5), and one of them had reported bioactivity (LLL). Charge mechanisms always predominated over the sieve effect, and best transmission was accomplished at pH values close to the peptides isoelectric points. Basic and neutral peptides with the highest scores were always the most transmitted. Membrane material had greater influence than NMWCO in determining peptide transmission. In order to obtain purified fractions rich in peptides with high bioactivity scores from serum albumin, polyethersulfone membranes (applicable to industrial scale) of 5 kDa MWCO should be used at basic pH values after pepsin digestion.

## Introduction

Bioactive peptides are defined as short amino acid fragments encrypted in the primary sequence of proteins that can confer health-related functions beyond basic nutritional benefits when orally administered^1^. Bioactive peptides similarity with endogenous hormones and neurotransmitters is the reason why they can affect the major body systems when ingested. Many beneficial health effects have been described for these molecules, e.g., antimicrobial, antioxidative, antithrombotic, antihypertensive and immunomodulatory^2^. One of the most common ways to obtain biopeptides from protein sources is enzymatic hydrolysis^3^. Enzymes offer the advantage of being highly specific in their mode of action, thus careful enzyme selection leads to different hydrolysates suitable for diverse applications^4^. Nevertheless, from all the peptides contained in a protein hydrolysate, not all of them will possess beneficial characteristics. Moreover, some might even be noxious or provide unwanted features^5^. Therefore, the need of a separation technology able to recognize small differences in charge, size, and hydrophobicity becomes evident.

Membrane systems can be used for separating the aforementioned complex mixtures. In fact, several authors have demonstrated the feasibility of high performance separations between proteins with very similar molecular size using membrane technologies^6,7^. In ultrafiltration (UF) and nanofiltration (NF), both molecular sieving mechanisms and charge effects are known to play a role on selectivity. However, many aspects of the separation mechanisms are still not clear^8^. In fact, existing mathematical models are unable to predict the separation processes, especially when filtrating complex mixtures such as protein hydrolysates. Peptide-peptide, membrane-peptide or polarized layer-peptide and membrane interactions are complex. Moreover, other phenomena such as hydrophobic interactions, aggregation or fouling processes complicate the prediction of the separation^9^.

Peptide isolation or purification from complex hydrolysates has been widely achieved using chromatographic methods^10,11^. However, the use of other technological approaches applicable at an industrial scale remains more scant^12^. Membrane technologies are low-energy processes that use mild operation conditions and are thus gentle with delicate molecules as proteins. They do not need the addition of chemicals and are very easy to scale-up^13^. There has been considerable interest in membrane filtration of protein hydrolysates during the last decade. However, the stress has been put in, for example, obtaining products with specific characteristics^14^ or filtration parameters optimization^15^; while little attention has been given to attempt to explain the mechanisms underlying the separation, except for^16,17^.

Besides, there is a dire need of finding industrial-applicable processes for obtaining valuable merchandises from food by-products, from both economical and environmental points of view^18^. Blood proteins are used mainly in food and feed industries as nutritional supplements, emulsifiers, stabilizers or clarifiers. Nevertheless, a big portion of blood is discarded as waste^19^. Bovine serum albumin (BSA), although a well known protein because of its ligand-binding capacity^20^, has not extensively been used with the purpose of obtaining bioactive peptides, in contrast with other well studied blood proteins such as haemoglobin^21,22^.

Therefore, this work had two main objectives: On the one hand, to present an exploratory procedure for obtaining a stream enriched in bioactive peptides from BSA, with future industrial applicability intended. Second, to assess the underlying mechanisms responsible for peptide transmission, with regards of membrane pore size and medium pH value.

## Material and Methods

### Materials

Pepsin (EC 3.4.23.1, from porcine gastric mucosa, activity ≥2500 U/mg protein) was from Sigma Aldrich (St. Louis, MO, USA). BSA isolated from cattle blood (purity >98%, fat <0.05%) was kindly supplied by Fedesa S.A. Laboratorios (San Luis, Argentina). Trifluoroacetic acid (TFA) was supplied by Merck (Darmstadt, Germany). Acetonitrile (ACN), formic acid and hydrochloric acid (HCl) were obtained from VWR (Barcelona, Spain). Sodium hydroxide was from Panreac (Barcelona, Spain).

### Peptic hydrolysis of BSA

BSA (3 g) was dissolved in ultrapure water (1 L) (MilliQ system, Millipore, Billerica, MA, USA) under constant agitation, using a magnetically stirred hotplate (MR Hei-standard, Heidolph, Schwabach, Germany). Solutions were allowed to reach the working temperature (37 °C) and the pH was adjusted to the enzyme optimum (2) using 2 M HCl. Hydrolysis reaction was started by the addition of pepsin at an enzyme to substrate (E/S) ratio of 4%. The pH values were maintained throughout digestion with an automatic pH regulator (pH burette 24 2 S, Crison, Barcelona, Spain). Fresh aliquots were collected and every 15 min after enzyme addition. In each sample and in the final hydrolysates (3 h), the enzyme was inactivated by raising the pH until 7^23^, using 0.1 N NaOH. Hydrolysates and samples were kept at −40 °C. Hydrolysis experiments were performed in duplicate.

### Calculation of the degree of hydrolysis (DH)

The DH was estimated using the Equation (1) ^24^.

1 $${\rm{DH}}( \% )=({{\rm{h}}/{\rm{h}}}_{{\rm{TOT}}})\times 100={[{\rm{A}}\times \text{NA}/(1-{\rm{\alpha }})\times {\rm{h}}}_{{\rm{TOT}}}{\times M}_{{\rm{p}}}]\times 100$$

where h is the number of peptide bonds broken (meq/g protein), and h_TOT_ is the total number of peptide bonds in the protein substrate (meq/g protein). For this work, the previously calculated value of 8.8 was used^25^. A is the acid consumption (mL), N_A_ is the normality of the acid (meq/mL), α is the average degree of dissociation of α-carboxylic groups and M_p_ is the mass of protein (g). In this study, α was calculated as in^26^, and took the value of 0.325.

Since the DH is defined as the percentage of peptide bonds cleaved during an enzymatic reaction, a DH of 100% means the complete degradation of a protein to free amino acids. Nevertheless, DH values are always less than 100% when enzymes with specificity to individual peptide bonds are used. BSA contains 582 peptide bonds, of which 147 are theoretical possible cleavage sites for pepsin (see Supplementary information 1). The program PeptideCutter (http://web.expasy.org/peptide_cutter/) was used to calculate pepsin cleavage sites. According to this, during BSA pepsinolysis, a maximum possible DH (DH_max_) of 25.26% can be achieved. Therefore, all the DH percentages were calculated relative to the theoretical DH_max_, as in Leeb, *et al*.^27^_._

### Membrane separation

#### Membrane rig

Two polyethersulfone (PES) membranes, each with a filtration area of 0.1 m^2^, were used. PES5 membrane (Millipore) had a nominal molecular weight cut-off (NMWCO) of 5 kDa, while PES1 (Sartorius, Goettingen, Germany) was of 1 kDa. Before first use, membranes were characterized with distilled water, and after every filtration run they were cleaned with 1 M NaOH and 2% H_3_PO_4_. The cleaning protocol was a 10 min rinse followed by 30 min recirculation at 40 °C at a constant transmembrane pressure (TMP) of 0.12 MPa for PES1 membrane and 0.20 MPa for PES5 membrane. The respective recirculation rates were 4 and 55 L/m^2^h. The cleaning procedure efficiency was checked by measuring the pure water flux (J_w_). J_w_ was always ≥95% recovered. Membranes were stored in 20% ethanol (PES1) or 0.1 M NaOH (PES5) under refrigeration (2–8 °C). Both membranes were installed within a Pellicon 2 mini cassette holder (88 cm^2^ & 0.11 m^2^, Millipore) connected to a 1 L jacketed glass tank reactor coupled to a thermostatic water bath for temperature control (Ultratherm, P Selecta, Barcelona, Spain). Fluid was pumped through the system by a GJ series 120 pump (I-Drive, Micropump Inc., Vancouver, WA, USA).

#### Hydrolysate fractionation

All membrane experiments were performed at fixed conditions of TMP (0.2 MPa) and temperature (37 °C), since membranes selectivity rather than parameter optimization was the interest of this work. Filtration was performed on full recirculation mode; at an average permeate flux (J_p_) of 20.7 ± 1.00 L/m^2^h for PES5 membrane and 7.8 ± 0.21 L/m^2^h for PES1 membrane. So as to study the role of pH dependent changes on the selective permeation of hydrolysate peptides, two pH values were assayed for each membrane (2 and 10), selected to represent different charge states of both the peptide mixture and the membrane surface. The pH was adjusted with 0.1 M HCl and 2 M NaOH. Before every filtration run the system was equilibrated for 15 min, and each pH value maintained for 1 h. All membrane experiments were performed in duplicate.

The observed transmissions (Tr_obs_) of individual peptides through the membranes were calculated using Eq. (2):

2 $${\rm{Tr}}( \% )=\frac{{{\rm{A}}}_{{\rm{Pi}}}}{{{\rm{A}}}_{{\rm{Hi}}}}\times 100$$

where A_Pi_ and A_Hi_ are the i peptide peak areas obtained from the UPLC chromatograms of each permeate and hydrolysate, respectively.

The theoretical transmissions (Tr_theo_) were also calculated according to Eq. (3)^28^:

3 $${\rm{Tr}}( \% )=[(1-{(\lambda \cdot (\lambda -2))}^{2})\cdot \exp (-0,7146{\lambda }^{2})]\times 100$$

with

$${\rm{\lambda }}={(\frac{{\rm{MW}}}{{\rm{MWCO}}})}^{0,4}$$

being MW the molecular weight of the peptide (Da) and MWCO the molecular weight cut off of the membrane.

The separation factors (S) between two peptides or group of peptides (_x/y_) were calculated as the ratio of mean Tr values (%) with Eq. (4):

4 $$\text{Sx}/y=\frac{{\sum }_{{\rm{i}}=1}^{{\rm{n}}}\,{\rm{Tri}}/{\rm{n}}}{{\sum }_{{\rm{j}}=1}^{{\rm{m}}}\,{\rm{Trj}}/{\rm{m}}}$$

where n and m are the number of peptides comprised in groups x and y respectively. This value (S_x/y_) represents the selectivity of the membrane to distinguish between specific peptides or groups of peptides.

### Peptide identification

RP-UPLC coupled to tandem mass spectrometry (RP-UPLC-MS/MS) was performed on a Dionex Ultimate 3000 RS UHPLC (Thermo Fisher Scientific, Waltham, MA, USA) connected on-line to a Bruker Impact II Q-ToF mass spectrometer (Bruker, Billerica, MA, USA). The column used was a Bruker Intensity Trio C18 (50 × 2.1 mm, 3um). Solvent A was composed of MilliQ water with 0.1% formic acid. Solvent B was composed of ACN with 0.1% formic acid. Gradient was 2% B for 1 min, from 2 to 35% B in 30 min, 35% B for 1 min, from 35 to 80% B in 4 min and 80% B for 1 min. The flux was 150 μL/min and the injection volume 2 μL. Separation was performed at 30 °C of temperature. Mass spectra were acquired in the positive ion mode using a 120 V fragmentation with a scan range of 50–3000 m/z. Nitrogen was used both as the drying gas at flow rate of 6.0 L/min and a temperature of 300 °C, and as the nebulizer gas at a pressure of 0.31 MPa. The capillary voltage was set at 4500 V. Only peaks with an intensity superior to the 10% of the most intense peak were considered for analysis, so as to avoid errors with sparse peptides. Identification analyses were all performed in duplicate. The ExPASy FindPept database (http://ca.expasy.org/tools/findpept.html) was used to identify the peptide masses obtained with the MS/MS analysis. FindPept performs a theoretical cleavage of a known protein by a known enzyme, displaying all possible peptide masses and their corresponding amino acid sequences resulting from both specific and non-specific cleavage. The program compares the experimental peptide masses to the theoretical ones, matching the similar and thus allowing identification. In this case, mass tolerance limit was set to 0.3 Da, and minimum peptide sequence length to 3 amino acids. The bibliography^29–31^ and BIOPEP database^32^ were searched to check if the identified peptide sequences had already been reported as bioactive. Peptide sequences GRAVY (average hydropathy score) and isoelectric points (pI) values were calculated with the ExPASy ProtParam tool (http://www.expasy.ch/tools/protparam-doc.html). The program Peptide Ranker (http://bioware.ucd.ie/~compass/biowareweb/Server_pages/peptideranker.php) was used to assess the potential bioactivity of the identified sequences^33^. Peptide Ranker assigns each sequence a score depending on the calculated probability of being bioactive.

Hydrolysates, retentates and permeates protein content was measured using a Pierce BCA protein assay kit (Thermo Fisher Scientific, Waltham, MA, USA).

### Statistical analyses

The results reported in this work are the average values of the experimental data, and the error bars indicate the relative experimental error. Analysis of variance (One-Way ANOVA) was carried out to assess whether the differences between groups or experiments were statistically significant, using the program StatPlus:Mac (AnalystSoft Inc., Walnut, CA, USA). The significance level was established for P < 0.05.

## Results and Discussion

### BSA hydrolysis with pepsin

**Figure 1:**
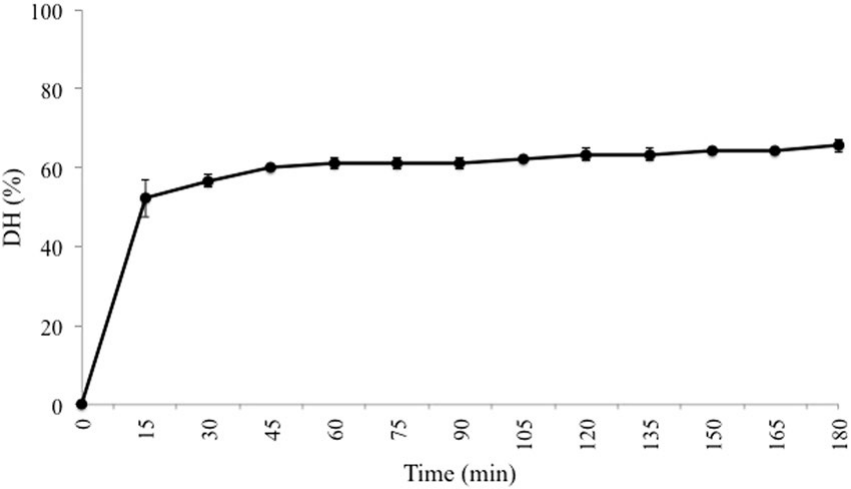
Hydrolysis curve for BSA (3 g/L) digestion with pepsin (4% E:S ratio) at 37 °C and pH 2. Data are presented as mean ± error.

BSA was hydrolysed with pepsin at the enzyme optimal conditions. The extent of protein hydrolysis was expressed as the DH, and monitored as a function of time (see Fig. 1). The DH values are relative to the DH_max_. As expected, the DH increased with hydrolysis time. However, during the first 15 min., the slope of the curve was very sharp, while over the rest of the process the DH increased steadily, reaching a plateau from 120 min on. Similarly, Lacroix and Li-Chan^34^ also found a slow down of pepsin activity after the first 30 min of hydrolysis. The maximum DH reached was a 65.54 ± 1.57% of the DH_max_ for the enzyme-substrate system. Although no bibliographical evidence was found of pure BSA pepsinolysis with the purpose of obtaining bioactive peptides in which the all the hydrolysate sequences were identified, several works about WPC and WPI hydrolysis with pepsin suggest BSA as a good substrate for the enzyme. For example, Kim, *et al*.^35^ and Peña-Ramos and Xiong^36^ found out that serum albumin was completely removed from SDS-PAGE gels after 30 min of pepsin hydrolysis of WPC solutions. Lacroix and Li-Chan^37^, whilst not obtaining a complete degradation, proved that BSA was the most digested protein amongst the individual whey proteins assayed (Alpha-lactalbumin (α-la), β-lg, BSA and Lactoferrin (LF)).

For explaining the high DH obtained, it should also be taken into account the low pH at which pepsin digestion was conducted. As pH decreases from neutrality to acidity, BSA domains are separated and helical content is lost, replaced by unstructured regions. Below pH 3.13, BSA acquires a linear bead form called “E state” (expanded)^38^. Pepsin preferentially cleaves peptide bonds with aromatic and hydrophobic amino acids^35^, which are normally hidden in the interior of the globular proteins^39^. With the unfolding, more of them become available for the enzyme^40,41^, and thus hydrolysis is enhanced. Likewise, the rate of proteolysis has been demonstrated to depend on the conformation of proteins^42^. Although protein hydrolysis was not complete, final hydrolysate RP-UPLC chromatograms (chromatograms not shown) evidenced no BSA peak. However, large peptide fragments of around 5–6 kDa were detected in the hydrolysate (see section 3.2).

### Peptide identification

For the sake of clarity, only peptides unequivocally identified and detected were considered. Peptide masses that could not be assigned to one single peptide sequence were 344.25, 360.21, 361.17, 375.22, 433.23, 462.27, 705.41 and 724.40, and thus were not considered. Peptides that were detected in any permeate but not detected in the hydrolysate were not considered either. A total of 19 peptide masses were excluded for this reason. Overall, 43 peptide masses were unambiguously assigned to peptide sequences. Of them, a 23.26% were acid, a 39.53% basic and a 37.21% neutral. Table 1 shows the identifications plus some relevant peptide characteristics. Only one of the identified peptides of Table 1 had previously been reported as bioactive, which is reasonable if we take into consideration the scarce works about peptide identification from BSA pepsinolysis. In fact, we have not found a complete report of the peptides yielded by pepsin digestion of BSA as the one we provide. Nevertheless, although IARRHPYF has not been reported as bioactive, a very close peptide sequence (IARRHPYFL) was reported as a neuropeptide^31^. The scores given by the program Peptide Ranker were used to assess peptides potential bioactivity (see Table 1). As it can be seen, a 23.25% of the peptides had bioactivity scores greater than 50%, indicator of probable bioactivity. The peptide with reported bioactivity is LLL, that, as expected, had a relatively good bioactivity score (0.57)^43^.

**Table 1:** Peptides identified by LC-MS/MS in the BSA peptic hydrolysate (3 g/L) and membrane permeates, plus relevant physiochemical characteristics. Peptide sequences are arranged by group type. Each group is arranged in descending order by potential bioactivity.

MW (Da)	Peptide sequence	Charge pH = 2^a^	Charge pH = 10^b^	Group^c^	GRAVY^d^	Bioactivity Score^e^
592.30	(F)/YAPEL/(L)	0.90	−2.50	A	−0.16	0.38
471.28	(A)/PELL/(Y)	0.90	−2.00	A	0.63	0.34
639.28	(E)/ACFAVE/(G)	0.90	−3.00	A	0.87	0.34
634.34	(A)/PELLY/(Y)	0.90	−2.50	A	0.24	0.32
596.29	(E)/YEATL/(E)	0.90	−2.50	A	0.02	0.13
424.21	(Y)/LYE/(I)	0.90	−2.50	A	−0.33	0.11
389.20	(E)/EQL/(K)	0.90	−2.00	A	−1.07	0.07
483.21	(E)/YEAT(L)	0.90	−2.50	A	−0.93	0.06
362.19	(E)/LTE/(F)	0.90	−2.00	A	−0.13	0.04
346.20	(F)/VEV(T)	0.90	−2.00	A	1.63	0.02
1059.58	(E)/IARRHPYF/(Y)	3.90	0.50	B	−0.75	0.73
731.42	(A)/WSVARL/(S)	1.90	0.00	B	0.60	0.68
517.28	(L)/HTLF/(G)	1.90	−1.00	B	0.68	0.59
401.29	(L)/LRL/(A)	1.90	0.00	B	1.03	0.56
1081.57	(L)/SQKFPKAEF/(V)	2.90	−1.00	B	−1.09	0.53
1075.52	(Y)/YANKYNGVF/(Q)	1.90	−1.50	B	−0.57	0.45
1122.65	(L)/PKLKPDPNTL/(C)	2.90	−1.00	B	−1.27	0.43
1238.58	(L)/YYANKYNGVF/(Q)	1.90	−2.00	B	−0.64	0.43
825.45	(E)/TYVPKAF/(D)	1.90	−1.00	B	0.19	0.30
1107.53	(E)/YSRRHPEY/(A)	3.90	−1.00	B	−2.59	0.28
550.33	(L)/IVRY/(T)	1.90	−0.50	B	0.73	0.20
893.48	(P)VSEKVTKC(C)	2.90	−2.00	B	−0.24	0.10
478.27	(L)/KTVM(E)	1.90	−0.50	B	0.38	0.09
801.52	(Q)/IKKQTAL/(V)	2.90	0.00	B	−0.27	0.09
1051.62	(L)/LKHKPKATE/(E)	4.90	−0.50	B	−1.68	0.08
838.48	(E)/HVKLVNE/(L)	2.90	−1.50	B	−0.27	0.05
1546.91	(L)/IVRYTRKVPQVST(P)	3.90	1.00	B	−0.34	0.05
414.20	(L)/FTF/(H)	0.90	−1.00	N	1.63	0.97
350.21	(L)/IAF/(S)	0.90	−1.00	N	3.03	0.82
514.23	(E)/YGFQ/(N)	0.90	−1.50	N	−0.60	0.79
358.27	(W)/LLL/(L)	0.90	−1.00	N	3.80	0.57
336.19	(F)/VAF/(V)	0.90	−1.00	N	2.90	0.56
423.22	(L)/GLAY/(P)	0.90	−1.50	N	0.98	0.46
330.20	(T)PTL/(V)	0.90	−1.00	N	0.50	0.35
713.42	(F)/AVEGPKL/(V)	1.90	−1.50	N	0.06	0.30
474.27	(D)RADL/(A)	1.90	−1.00	N	−0.60	0.26
382.20	(D)YLS(L)	0.90	−1.50	N	0.57	0.24
940.48	(E)/TYVPKAFD(E)	1.90	−2.00	N	−0.28	0.17
488.31	(Y)/AVSVL/(L)	0.90	−1.00	N	2.64	0.12
700.42	(L)/LPKIET(M)	1.90	−1.50	N	−0.23	0.10
417.27	(A)/VSVL/(L)	0.90	−1.00	N	2.85	0.08
688.43	(F)/VEVTKL/(V)	1.90	−1.50	N	0.68	0.05
533.29	(L)/VVSTQ/(T)	0.90	−1.00	N	0.68	0.03

^a,b,d^Calculated with the Expasy Molecular Biology Server (http://www.expasy.org/).

^c^Calculated as in Lapointe, *et al*.^44^.

^e^Calculated with Peptide Ranker *in silico* tool (http://bioware.ucd.ie/∼compass/biowareweb/).

### Membrane fractionation

#### Peptide transmissions

**Figure 2:**
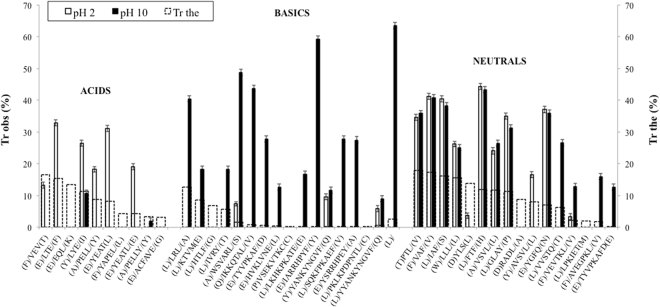
Observed and theoretical transmission values across PES1 membrane for BSA peptic hydrolysate identified peptides. Data are presented as mean ± error.

**Figure 3:**
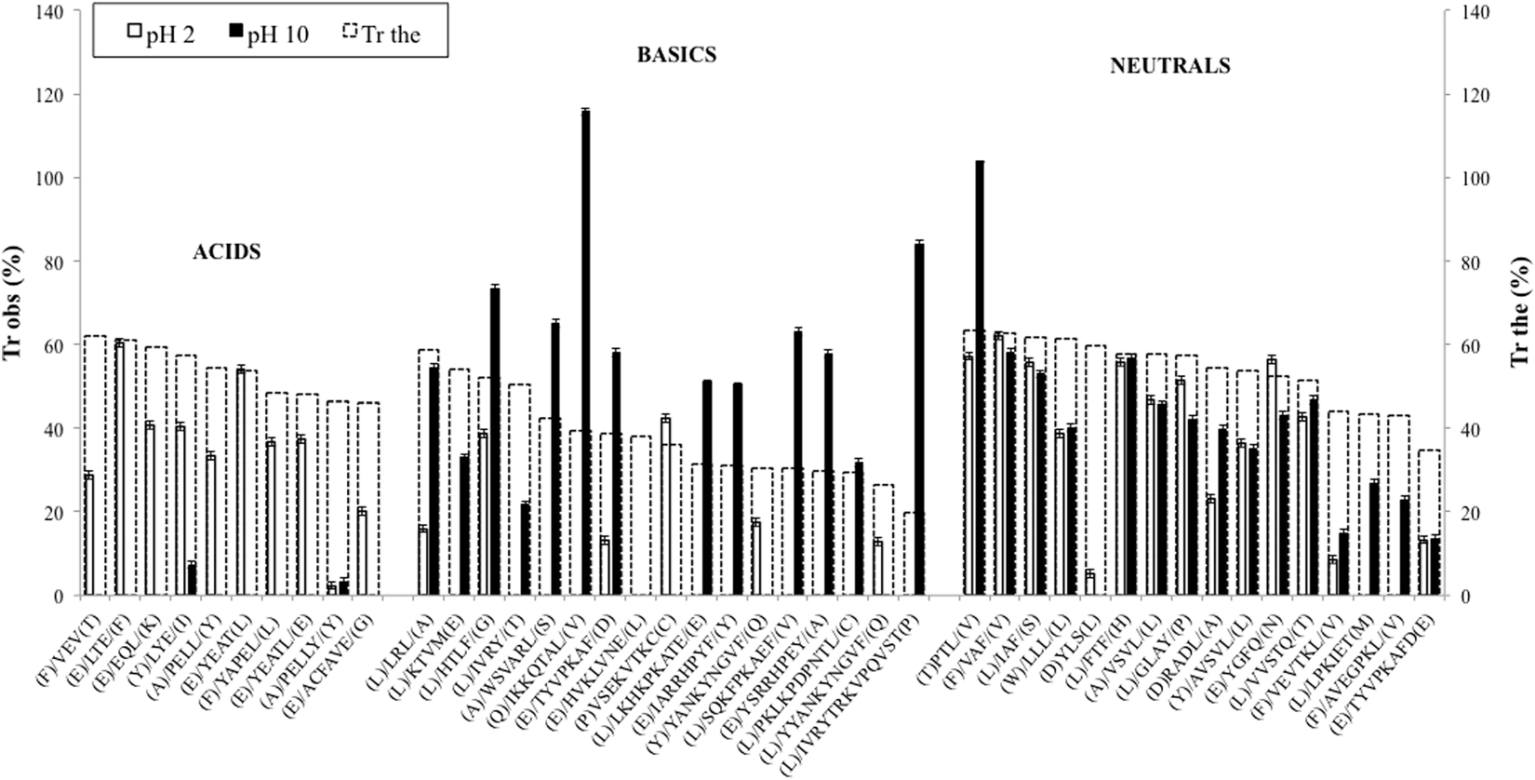
Observed and theoretical transmission values across PES5 membrane for BSA peptic hydrolysate identified peptides. Data are presented as mean ± error.

Figures 2 and 3 display Tr values corresponding to filtration experiments. Solid bars represent Tr_obs_, while dotted bars reflect Tr_the_. Peptides were divided in three groups according to their pI values^44^: acid (pI < 5.0), basic (pI > 8.0) and neutral (5.0 < pI < 8.0).

In general, it can be seen that Tr_obs_ were different from the calculated Tr_the_ for both PES1 and PES5 membranes, revealing the influence of other parameters apart from peptide size (see section 1). Tr values were generally higher than expected for PES1 membrane, while for PES5 membrane were both higher or lower. Additionally, Tr_obs_ changed greatly with pH, and the variation was markedly different for each peptide group. Similarly, when filtrating a tryptic β-lg hydrolysate with a 5 kDa polyethersulfone membrane, Fernández, *et al*.^17^ also realised that peptide Tr was mainly governed by charge mechanisms. Membrane NWCO had a relevant influence too, as Tr values were, in general, higher for PES5 membrane in the majority of the cases. At pH2, PES5 Tr_obs_ overall values were 2.23 times higher than PES1 Tr_obs_ values; and at pH10, the factor was 1.80. Despite the great difference in NMWCO, peptides transmitted across both membranes were the same, indicating that membrane material rather than pore size defines transmission.

In order to provide rough guidance about protein concentration in both membrane streams, a BCA assay was performed, indicating a protein content of 2.71 and 0.23 mg/mL in retentate and permeate streams respectively.

#### Acidic peptides

Speaking about general trends, acidic peptides were far more transmitted at acid pH value (30.96% on average) than basic pH value (5.85% on average), for both membranes. In fact, only two peptides were detected in the basic permeate streams (LYE and PELLY), and with very low Tr values, of less than 10% in all cases. This as well (see section 3.3.2) stresses the higher influence of charge state above other parameters, in this case ionic strength. In order to raise the pH from 2 to 10, base was added, and thus ionic strength increased. It is well known that an increase in salt concentration decreases electrostatic interactions^45^. This usually leads to better Tr of the peptides with the same sign as the membrane. In this case, despite the smaller radius and the less intense repulsive forces, acidic peptides were not transmitted at basic pH values, except for LYE and PELLY. With regards to the charge effect, at pH values <than 4, the membrane loses its original negative charge^46^, while acidic peptides are very near of their pI (see Table 1). Consequently, their charge is very small and thus they can be easily transmitted^17^, due to the lack of electrostatic repulsive forces. PELLY showed very low Tr values, and almost no difference between pH values. However, PELL, while only differing in one amino acid, showed much higher Tr values. At pH10, PELLY is even more charged than PELL. These results do not have to be contradictory, since Pouliot, *et al*.^47^ showed that two peptides that only differed in one amino acid could be transmitted in a different way. Also, acidic peptides were all more transmitted through the membrane with higher MWCO, with no exceptions (Average Tr of 23.47% for PES1, while 35.45% for PES5). Regarding Tr_the_, all acidic peptides were less transmitted than expected for PES5 membrane, but more transmitted that expected for PES1 membrane, highlighting the influence of charge above size. Acidic peptides did not have good bioactivity scores, being the highest a 0.38% for peptide YAPEL. Thus, a filtration pH of 2 is not a good choice when trying to obtain permeates rich in bioactive peptides from a BSA peptic hydrolysate.

#### Basic peptides

In general, basic peptides were far more transmitted at basic pH values. As exceptions, VSEKVTKC was only transmitted at pH2 through PES5; and YANKYNGVF and YYANKYNGVF were more transmitted at pH2 than at pH10. The fact that both similar peptides had the same behaviour points towards a phenomenon rather than an experimental error. Moreover, these are also peptides with a very hydrophobic peptide on one side. Therefore, hydrophobic interactions with the membrane retained them and thus worked against Tr. As acidic peptides, almost all of the basic ones were more transmitted through the bigger NMWCO membrane, except HVKLVNE, IARRHPYF and YANKYNGVF/YYANKYNGVF.

Basic peptides were the group that had, on average, greater Tr values through both membranes at pH10. Apart from the aforementioned charge effect, the greater ionic strength could have added a synergistic effect, decreasing the intensity of the electrostatic charges and reducing peptides radius.

IKKQTAL was the only detected peptide with Tr higher than 100% (except PTL, from the neutral group). As Tr values are calculated from HPLC chromatograms peak areas, some small errors can be expected. What is clear is that this peptide had a very high Tr, consequence of its zero net charge at pH 10. Nevertheless, LRL and WVASRL also had 0 net charge and had lower Tr values. However, the latter were quite hydrophobic peptides, while the former quite hydrophilic. It has been described that hydrophobic amino acids can establish hydrophobic interactions with PES membranes^48^, so these interactions retained both hydrophobic peptides, lowering their Tr values.

Some basic peptides had very good bioactivity scores, such as IARRHPYF, WSVARL, HTLF or LRL. Interestingly, IARRHPYF (115.7%) was the most transmitted peptide, followed by HTLF (73.3%) and WSVARL (65.0%), across PES5 membrane at pH10. Therefore, filtration of BSA peptic hydrolysates at basic pH values through 5 kDa MWCO PES membranes is evidenced as a good strategy to enrich permeates in bioactive peptides.

#### Neutral peptides

Neutral peptides were in general more transmitted through the higher MWCO membrane. In this case, pH influence on transmission was very low, since almost all peptides were equally transmitted at both pH values. It can be seen in Table 1 that, for both pH values, the great part of the neutral peptides had similar absolute charge values (around 1), taking into account that charge sign was the opposite. Exceptions were PTL, which was much more transmitted at pH10; and YLS, that had very low Tr values. Some peptides were not transmitted at pH2 through one or both membranes, such as RADL, AVSVL, VVSTQ, LPKIET and AVEGPKL, with no apparent reason for this behavior.

Several neutral peptides also had very high bioactivity scores, and were also some of the best transmitted, such as FTF (best Tr_obs_ = 57.8%), IAF (61.9%) and YGFQ (52.2%). Neutral peptides presented two advantages: they had overall Tr values very similar to the basic group and thus relatively high, and they had the same behavior at both pH values assayed. Further *in vitro* and even *in vivo* bioactivity assays would be interesting for neutral and basic peptides with high bioactivity scores that are transmitted in high percentages across PES membranes.

#### Separation factors

Table 2 summarizes the separation factors calculated for each peptide group at every set of conditions assayed. Overall, all the values were relatively high if compared with other works of hydrolysates filtration in which separation factors were similarly calculated. For instance, Fernández, *et al*.^17^ obtained separation factors that ranged from 0.9 to 291.8. In this work, the pH-membrane combination that presented the worst separation factors was pH2 and PES5 membrane; and the best pH10 and PES5 membrane. Fernández, *et al*.^17^ also obtained better separation factors at pH 10.

**Table 2:** Separation factors for all the combinations membrane-pH assayed.

	pH2		pH10	
	PES1	PES5	PES1	PES5
A/N	135.92	102.52	1691.30	3765.36
A/B	1049.77	428.95	1962.46	4189.63
B/N	1426.88	418.43	116.03	111.27

A = acid peptides; B = basic peptides; N = neutral peptides.

In order to achieve a good separation between acid and neutral peptides or acid and basic peptides, the latter combination would be the best choice as it had the highest value for both cases. However, when trying to separate basic and neutral peptides PES1 membrane at a pH value of 2 worked better. Interestingly, the best selectivity of the PES5 membrane at pH values of 10 is coincident with the enrichment in peptides with high bioactivity scores at those conditions.

In general, neutral and basic peptides were separated with more difficulty than each of them from the acid group. It should be taken into account that, although acid and neutral groups average molecular weight (MW) was very similar (493.85 and 504.04 kDa respectively), basic peptides were bigger in general (901.26 kDa), and thus sieving effect had an additional influence when separating this last group. This is why acid/basic separation was always higher than acid/neutral separation at all the conditions tested.

## Conclusions

BSA at pH2 is a good substrate for pepsin, since a high DH value was achieved (77.68 ± 1.86% of the DH_max_). Besides, BSA pepsinolysis yields a 23.25% of peptides with high bioactivity scores (more than 0.50), and at least one peptide with confirmed bioactivity (LLL).

Regarding peptide Tr values, charge was the predominant mechanism responsible for transmission rather than size, since Tr_obs_ were different from Tr_the_, and Tr_obs_ were different for each peptide group at each pH value assayed. Within each group, higher Tr values were achieved at the pH value that was nearest to the group pI. However, membrane MWCO also had an important influence, as peptide overall Tr values trough PES5 membrane were approximately twice PES1 Tr values. Membrane material had greater influence than NMWCO in determining peptide transmission. Acidic peptides were more transmitted at pH2 and through PES5 membrane, but had low bioactivity scores. Consequently, filtration at pH2 is not recommended for obtaining permeates enriched in potentially bioactive peptides. Basic and neutral peptides, on the contrary, had better bioactivity scores, and were best transmitted at pH10 with PES5 membrane. Interestingly, most transmitted peptides were the ones that had highest bioactivity scores. Therefore, filtration of BSA peptic hydrolysates with a PES5 membrane at pH10 is evidenced as a good strategy, applicable to industrial scale, to obtain permeates enriched in neutral and basic peptides with very high bioactivity scores. In agreement with the latter, best separation factors were achieved at pH10 with PES5 membrane. In general, acid peptides were more easily separated from the other groups than neutral and basic between them. A new line of bioactivity testing research is suggested with peptides best transmitted and with high bioactivity scores.

## Electronic supplementary material

Supplementary information
