# Supplementary material for: Utilization of blood by-products: An in silico and experimental combined study for BSA usage
Source: Sci Rep. 2017 Dec 8;7:17250. doi: 10.1038/s41598-017-17029-2 (PMC5722935; doi:10.1038/s41598-017-17029-2)
Supplement: Supplementary file 1 — Supplementary information [file 41598_2017_17029_MOESM1_ESM.pdf]

Title: Utilization of blood by-products: An *in silico* and experimental combined study for BSA usage

Short title: An *in silico* and experimental combined study for BSA utilization

Authors: Fátima Arrutia[1*], Rebeca Fernández[1], Carlos Menéndez[2], Ulises A. González[2] and Francisco A. Riera[1].

1: Chemical Engineering and Environmental Technology Department, University of Oviedo, Julián Clavería, 8, 33006, Oviedo, Asturias, Spain.

[2] Research Institute in Chemical Technology (INTEQUI)-CONICET, Faculty of Chemistry, Biochemistry and Pharmacy, Universidad Nacional de San Luis (UNSL), Ejército de los Andes 950, 5700 San Luis, Argentina.

* Corresponding author e-mail: farrutia@gmail.com

PeptideCutter

The sequence to investigate:

```
        10         20         30         40         50         60
DTHKSEIAHR FKDLGEEHFK GLVLIAFSQY LQQCPFDEHV KLVNELTEFA KTCVADESHA

        70         80         90        100        110        120
GCEKSLHTLF GDELCKVASL RETYGDMADC CEKQEPERNE CFLSHKDDSP DLPKLKPDPN

       130        140        150        160        170        180
TLCDEFKADE KKFWGKYLYE IARRHPYFYA PELLYYANKY NGVFQECCQA EDKGACLLPK

       190        200        210        220        230        240
IETMREKVLT SSARQRLRCA SIQKFGERAL KAWSVARLSQ KFPKAEFVEV TKLVTDLTKV

       250        260        270        280        290        300
HKECCHGDLL ECADDRADLA KYICDNQDTI SSKLKECCDK PLLEKSHCIA EVEKDAIPEN

       310        320        330        340        350        360
LPPLTADFAE DKDVCKNYQE AKDAFLGSFL YEYSRRHPEY AVSVLLRLAK EYEATLEECC

       370        380        390        400        410        420
AKDDPHACYS TVFDKLKHLV DEPQNLIKQN CDQFEKLGEY GFQNALIVRY TRKVPQVSTP

       430        440        450        460        470        480
TLVEVSRSLG KVGTRCCTKP ESERMPCTED YLSLILNRLC VLHEKTPVSE KVTKCCTESL

       490        500        510        520        530        540
VNRRPCFSAL TPDETYVPKA FDEKLFTFHA DICTLPDTEK QIKKQTALVE LLKHKPKATE

       550        560        570        580
EQLKTVMENF VAFVDKCCAA DDKEACFAVE GPKLVVSTQT ALA
```

The sequence is 583 amino acids long.

Available enzymes

The enzyme(s) that you have chosen:

- Pepsin (pH>2)

You have chosen to display all possible cleaving enzymes.

These enzymes cleave the sequence:

Name of enzyme	No. of cleavages	Positions of cleavage sites
Pepsin (pH>2)	147	13 18 19 21 23 24 26 27 29 30 31 35 42 45 46 48 49 65 68 70 73 74 79 80 84 101 102 103 112 121 122 125 126 132 136 137 139 148 153 154 155 156 159 160 163 164 176 178 188 197 204 205 209 212 218 222 227 232 233 236 237 249 250 259 261 262 273 274 281 283 307 308 317 325 326 328 329 330 331 332 333 340 344 345 346 348 351 355 356 369 372 373 375 376 378 385 386 393 394 396 397 399 400 401 402 405 406 410 422 428 450 451 452 453 454 455 456 459 461 462 479 480 487 489 495 500 504 505 507 508 515 527 528 530 531 532 542 543 549 550 552 553 566 567 574 581 582

At these positions the following enzymes cleave:

- Please note that the size of the peptides are calculated as if **all chosen enzymes were present**
